# Feasibility of Self-Monitoring Rheumatoid Arthritis With a Smartphone App: Results of Two Mixed-Methods Pilot Studies

**DOI:** 10.2196/20165

**Published:** 2020-09-21

**Authors:** Bart F Seppen, Jimmy Wiegel, Merel J L'ami, Sharon Duarte dos Santos Rico, Fabio S Catarinella, Franktien Turkstra, Maarten Boers, Wouter H Bos

**Affiliations:** 1 Amsterdam Rheumatology and Immunology Center Reade Amsterdam Netherlands; 2 Department of Rheumatology VU Medical Center Amsterdam UMC Amsterdam Netherlands; 3 Brightfish Ltd Amsterdam Netherlands; 4 Department of Epidemiology & Biostatistics Amsterdam Public Health Vrije Universiteit Amsterdam, Amsterdam UMC Amsterdam Netherlands

**Keywords:** mHealth, eHealth, patient-reported outcome, smartphone app, rheumatoid arthritis, self-monitoring, telemonitoring, mobile phone

## Abstract

**Background:**

Several mobile apps that monitor symptoms of rheumatoid arthritis (RA) exist, but a recent systematic review indicated that high-quality apps are lacking. When patients self-monitor their own disease with patient-reported outcomes (PROs) and self-initiate care at the right moment, it may be possible to reduce the frequency of their clinic visits, which would reduce health care burden and costs. We developed an app, that is, the MijnReuma Reade app, for this purpose and performed 2 pilot tests with weekly self-monitoring.

**Objective:**

The primary objective of this study was to design, develop, and evaluate the usability, satisfaction, and usage of the MijnReuma Reade app—an app that allows patients with RA to monitor their own disease. The secondary objective was to review the patients’ perspectives on app usage and its intended purpose.

**Methods:**

This app was designed in collaboration with patients with RA, rheumatologists, and information technology experts. Two 1-month pilot studies were performed, after which satisfaction (0-10 scale), usability (system usability scale, 0-100), and usage (proportion of completed questionnaires) of this app were assessed. After the second pilot study, semistructured interviews were performed to determine patients’ perspectives and the promoters and barriers of app usage.

**Results:**

In the first and second pilot study, 42 and 27 patients were included, respectively. Overall, the patients were satisfied (medians, 8 and 7) and found the app usable (mean system usability scores, 76 and 71) in pilot studies 1 and 2, respectively. App usage declined over time in both the pilot studies; 61% (17/28) and 37% (10/27) of the patients who disclosed their usage statistics completed the final weekly questionnaire in pilot study 1 and pilot study 2, respectively. Approximately 81% (25/31) of the patients indicated they would like to skip hospital visits if the self-monitored disease activity is low. In the semistructured interviews, technical problems, internal resistance (respondent fatigue, the app reminded them of their disease), and a lack of symptoms were identified as barriers for usage. Patients reported that “experiencing more grip on their disease” and “improved communication with their physician” were promoters for usage. Patients reported that pain positively mediated usage, that is, more pain promoted and less pain discouraged app usage.

**Conclusions:**

This study illustrates the feasibility of the MijnReuma Reade app that enables self-monitoring of the disease activity in patients with RA with the overarching aim to allocate clinical consultations according to need. Satisfaction with the app and usability of the app were found to be high; however, app usage declined over time. Patients acknowledged the potential of the app to self-monitor their own disease and would like to be able to skip clinic visits if the monitored disease activity is low. To evaluate this strategy, a randomized controlled trial is underway.

## Introduction

eHealth—the health care practice supported by electronic processes and communication—is an upcoming theme in medicine [1]. One of the quickly developing fields within eHealth is mobile health (mHealth) care. mHealth promises to provide medical support for patients through mobile devices such as smartphones or tablets [2]. In rheumatology, we can use mHealth to enable patients to self-monitor their own conditions with patient-reported outcomes (PROs) [3-5], which in turn could support self-initiated care [6]. Most patients visit their rheumatologist every 3-6 months to evaluate disease activity [7]. The value of many of these consultations might be low, as many patients, at least in the affluent societies, have minimal disease activity [8]. Furthermore, due to the capricious nature of rheumatoid arthritis (RA), clinically relevant flares that occur between visits may be missed when patients visit the outpatient clinic according to predetermined schedules [9]. With mHealth, it is possible to monitor disease activity frequently [4,10], and thus, it may improve the clinical management of patients by better allocating clinical consultations according to need [11,12]. Several mobile apps that self-monitor disease activity already exist [13]. However, multiple studies have indicated that there is still a lack of high-quality apps for self-monitoring RA disease activity [13-15]. The quality of the apps can only be confirmed after a thorough and repeated clinical evaluation. This paper reports the development of an app to self-monitor RA disease activity and the results of 2 mixed-methods pilot studies. The research questions in the pilot studies were as follows:

1. Primary research question: Is it feasible to let patients with RA self-monitor their disease with the use of the MijnReuma Reade app, in terms of satisfaction, usability, and app usage?
2. Secondary research question: What are the perspectives of the patients regarding the app and self-monitoring with the purpose of reducing unnecessary consultations?

## Methods

### Setting and Subjects

The pilot studies were performed at Reade, a center for rheumatology and rehabilitation in Amsterdam. The city of Amsterdam along with its surroundings is an ideal setting for mHealth studies, as network coverage is excellent, download speeds rank 6th worldwide, and 87% of the adult population in Amsterdam own a smartphone [16-18]. In 2015, Reade started improving its information technology infrastructure [19]. The first goal was to digitize PROs. This facilitates electronic questionnaire assessment and integration of clinical data such as laboratory results, radiology reports, and severity scores with the PROs. Reade has now set the aim to extend the electronic PRO infrastructure to outside the walls of the hospital. In order to do this, we built an app that allows patients to access and complete PROs. Patients were informed about the app and invited to participate in the pilot studies during regular outpatient clinic consultations by their treating rheumatologists. When patients indicated an interest to participate in these pilot studies to their rheumatologist, they were called by a researcher (SR). Interested patients were included if they met the following criteria: diagnosed with RA, 18 years or older, able to read Dutch, and own a smartphone or tablet with an Android or iPhone operating system. No exclusion criteria were set. All patients signed informed consent.

### Study Design

Patients were asked to download the app from the app store and complete a questionnaire in the app every week for 4 weeks. In pilot study 1, the weekly questionnaire comprised the full multidimensional Health Assessment Questionnaire (HAQ, including an RA disease activity index and symptom list). In pilot study 2, we downsized the weekly questionnaire to Routine Assessment of Patient Index Data 3 (RAPID3) with additional questions regarding fatigue, sleep, morning stiffness, anxiety, stress, and social participation as found in the HAQ-II. After 4 weeks, a questionnaire was sent to the patients through email to evaluate the usability, satisfaction, and qualitative outcomes. Patients who stopped the study or never installed the app were not sent the final questionnaire. Technical problems reported by patients were recorded in an Excel logbook. The local Reade/Slotervaart hospital medical ethical committee issued a waiver for this study.

### Outcome Measures and Data Collection

The primary outcomes of the pilot studies were satisfaction, usability, and app usage. Overall satisfaction was measured on a 10-point Likert scale (eg, How would you rate the app?). Alternatively, patient satisfaction was measured with the Net Promoter Score (NPS); this tool allows patients to rate the extent to which they would recommend the use of the app to a friend or colleague [20]. This tool, often used in customer loyalty research, predicts how likely a customer would recommend a product on an 11-point Likert scale. Patients who scored the app 9 or 10 were considered as promoters of the app, 7 or 8 were considered as neutrals or passive enthusiasts, and 0-6 were considered as detractors. Grouping patients into these 3 categories, that is, promoters, passive enthusiasts, and detractors, provides a simple intuitive scheme that accurately predicts the users’ behavior (ie, in business: the repurchase rate). The NPS is calculated by subtracting the proportion of critics from the proportion of promoters. Usability was evaluated with the system usability scale. The system usability scale has proved to be a valuable evaluation tool since it is highly robust and reliable [21,22]. The average system usability scale score is 68; a mean score of 52 indicates OK usability and 72 indicates good usability [23]. The final questionnaire included 2 additional questions regarding usability rated on a 10-point Likert scale ranging from “definitely agree, 10” to “definitely disagree, 1” (eg, “I use every function in the app” and “I think the explanation on how the app works is clear”). As proxy for app usage, we used the weekly response rate for RAPID3. All outcomes were presented in descriptive statistics.

### Patients’ Perspectives in Pilot Study 1

To assess the patients’ perspectives, the final survey included statements regarding the app, its purpose and possible features, and a free text field. The statements were adapted from Vorrink et al [24] for use in rheumatology and are presented in Multimedia Appendix 1. Patients were presented 17 “overall feedback” statements and 9 “privacy statements,” which they could score on a 10-point Likert scale (ranging from 1=definitely do not agree to 10=definitely agree). In the next section of the questionnaire, patients could (optionally) provide their opinion on what aspects of the app were unnecessary, unclear, or could be improved and what sections were useful and clear in a free text field.

### Semistructured Interviews in Pilot Study 2

Patients in the second pilot study were asked to take part in a semistructured interview to explore their perspectives on the app, its intended purpose, and app usage. Patients were purposefully selected to form a varied group that included patients who frequently used the app, patients who discontinued use during the study, and patients who did not use the app more than once. The recruitment of patients continued until data saturation. One team member (BS) conducted telephone interviews (15 minutes) in November and December 2018. BS was not previously known to the patients and was not involved in the feasibility studies. Patients gave verbal consent for audio recording. Patients’ experiences of using the app were explored following a 7-question interview guide (Multimedia Appendix 2). The questions were in part derived from themes in the mobile app rating scale and in part through discussion between authors BS and WB [25]. Questions were intended to guide the conversation, rather than to be prescriptive. The interviewer responded to patients’ comments and encouraged them to talk freely to maximize informative comments. All interviews were audiotaped and transcribed (BS). Patients’ perspectives on the app, app usage, and its intended purpose were thematically coded. The coding and thematic analysis were performed by BS; subsequently, 2 investigators (BS/WB) discussed the data. Illustrative comments were selected to illustrate the patients’ perspectives and the identified barriers and promoters of usage.

### App Development

The development and evaluation of the app were carried out in 3 distinct phases according to the Medical Research Council guidance for developing and evaluating complex interventions [26]. The 3 phases were as follows: (1) setting design requirements, building the prototype, and the first evaluation, (2) improvement of the prototype and re-evaluation, (3) further improvement of the app and a randomized controlled trial. Phase 3 has been described previously [6].

### Design of the Prototype

The prototype was developed in 2016. As recommended [14], this was done by a collaboration of patients (enthusiastic volunteers), nurses, rheumatologists, and information technology experts (Brightfish Ltd). The following design requirements were set.

1. Integration of a validated PRO.

2. Short weekly 5-minute questionnaires.

3. High usability and user satisfaction.

4. Multiplatform (native iPhone, native Android, and web-based operating systems).

5. Provision of helpful information for patients about RA.

6. Integration with the electronic medical record.

**Figure 1:**
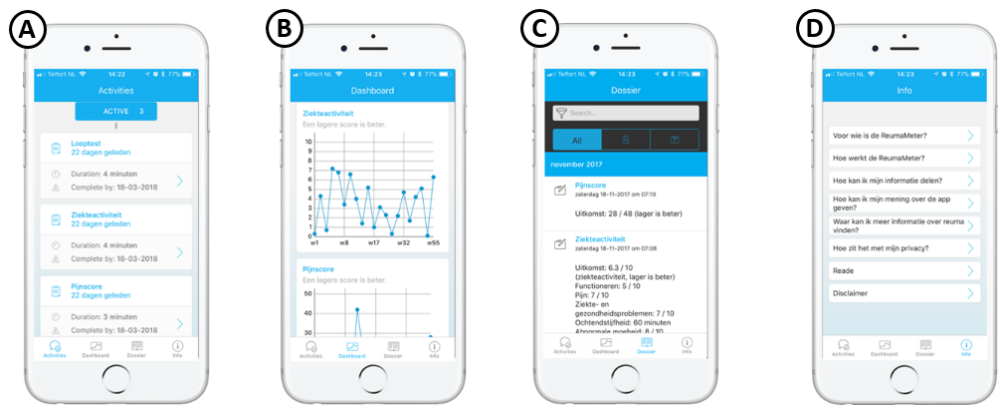
The MijnReuma Reade App prototype. A. The activity module where due questionnaires are found. B. The dashboard module that displays several disease outcomes over time. C. The dossier module that displays the numerical answers given to the different questionnaires. D. The information module that provides information on the app and rheumatoid arthritis. English translation ("Dutch translation"); walking test ("Looptest’’); disease activity ("ziekte-activiteit’’); pain score ("pijn score’’).

A prototype MijnReuma Reade app was built by an information technology company (Figure 1). This prototype met design requirements 1-5. As a validated PRO, the multidimensional RAPID3-HAQ-II was chosen by the study team [27]. In the app, all the domains of disease activity are displayed in illustrative graphs over time [28,29]. BrightFish developed the interface to be easy to use and intuitive. Before the initiation of the pilot study, we performed a small pretest. The goal was to explore areas of confusion and areas to improve user experience. Fifteen volunteers completed the questionnaire in the app, while being observed by a rheumatologist (WB) and an information technology expert. No areas of confusion or problems that required immediate repair were noted; therefore, we concluded that the app could be used in the first pilot study. After the first pilot study, a new phase of development took place. First, we shortened the questionnaire after pilot study 1 to meet the 5-minute requirement. Second, the app was integrated with the electronic medical record. Patients were now able to see their laboratory results and appointments. Furthermore, the filled out questionnaires were now visible in the patients’ medical files at Reade. This version of the app was used in the second pilot study.

## Results

### Patient Characteristics

For pilot study 1 and 2, 42 and 27 patients signed informed consent, respectively; 24 of the 27 patients in the second pilot study had also participated in the first pilot study. Patient characteristics are summarized in Table 1. During the first pilot study (n=42), 5 patients dropped out (12%), leaving 37 patients. The reasons for dropout were as follows: never downloaded or used the app (n=2) and technical problems with (n=1) or without (n=2) direct relationship to the app (n=2). Of the 37 patients who completed the first pilot study, 31 patients filled the questionnaire to evaluate the app, while 6 patients did not respond after several reminders. Three patients did not provide their app ID in the questionnaire; the app ID was necessary to request the usage statistics from the software company. Thus, we analyzed the app usage of 28 patients. In the second pilot study, 2 patients never installed the app, 1 decided not to participate after consenting, and 5 did not complete the final questionnaire, leaving 19 patients for analysis.

**Table 1:** Patient characteristics.

Patient characteristics	Pilot study 1, n=42	Pilot study 2, n=27
Age (years), mean (SD)	54 (13)	52 (11)
Females, n (%)	36 (86)	21 (78)
Baseline disease activity score in 28 joints, mean (SD)	2.88 (1.28)	2.6 (1.48)
Disease duration (years), median (25th percentile, 75th percentile)	9 (4,13)	7 (3,8)
Biological use (yes), n (%)	27 (64)	16 (59)

### Primary Outcomes

The primary outcomes are summarized in Table 2. Overall, the app was rated with satisfaction scores of 8.0 (IQR 7.0-9.0) and 7.0 (IQR 6.0-8.0) in the first and second pilot study, respectively. In the first pilot study, the NPS of the app was –9 (9/31 [29%] promoters, 10/31 [32%] passives, and 39% [12/31] detractors); in the second pilot study, the NPS was neutral (37% [7/19] promoters, 26% [5/19] passives, and 37% [7/19] detractors). The completion rates of the weekly in-app questionnaires declined over time in both pilot studies. In the first and second pilot study, the completion rates declined from 100% (28/28) and 78% (21/27) in week 1 to 61% (17/28) and 37% (10/27) in week 4, respectively.

**Table 2:** Primary outcomes of the pilot studies.

Outcomes		Pilot study 1	Pilot study 2
Overall satisfaction score, median (25th percentile, 75th percentile)^a^		8 (7,9)	7 (6,8)
**Net promoter score^b^**			
	Total score	–9	0
	Promoters, n (%)	9 (29)	7 (37)
	Detractors, n (%)	12 (39)	7 (37)
**Usability**			
	System usability score, mean (SD)^c^	76 (15)	71 (20)
**Usage^d^**			
	Week 1, n (%)	28 (100)	21 (78)
	Week 2, n (%)	26 (93)	11 (41)
	Week 3, n (%)	21 (75)	11 (41)
	Week 4, n (%)	17 (61)	10 (37)

^a^Scale of 1-10. The higher the score, the higher the satisfaction.

^b^Pilot study 1, n=31; Pilot study 2, n=19.

^c^Scale of 0-100.

^d^Percentage of weekly questionnaires that were completed in the app. Pilot study 1, n=28; Pilot study 2, n=27.

### Secondary Outcomes

#### Qualitative Outcomes of Pilot Study 1

Patient opinions on the feedback statements have been shown in a heat map (Multimedia Appendix 3). In the open feedback fields, several patients reported that the HAQ-II was too long (over 5 minutes), which meant that it did not meet the set design requirements. No other issues with the app were reported. Patients indicated that the graphs (displaying outcomes over time) were “useful” and that “the interface was clear.”

#### Qualitative Outcomes of Pilot Study 2: Semistructured Interviews

In general, the app was described as “clear,” “easy to use,” and “user friendly.” Patients acknowledged that the app had the potential to improve insight in disease activity over time and that it could help to reduce the burden of unnecessary outpatient clinic visits in time. Usage of the app varied between the patients. When asked to state reasons for not using the app, the following barriers for app usage were identified: technical problems, internal resistance (respondent fatigue, the app reminded them of their disease), and a lack of symptoms. We also identified 3 promoters for app usage: experiencing more grip on the disease, better communication with the physician, and an increase in disease activity. It can be noted that symptoms anecdotally play a modulating role in usage, as more symptoms induce usage whereas a lack of symptoms functions as a barrier for usage. A total of 5 illustrative quotes were chosen, which are presented in Table 3.

To optimize the app, several patients indicated that they desired an open field to disclose some notes with their submitted questionnaires, as they sometimes felt that the questionnaire did not fully capture their symptoms or that symptoms might be caused by something else. Other desires were a medication alarm/reminder, touch ID to log in, more graphs to display outcomes over time, a two-way chat function, or a change in questionnaires. Conflicting opinions were given regarding the addition of game-like elements to the app. Some opinions were positive such as “good, if it helps to me to fill out more questionnaires,” and “fun, if I can win something,” while some opinions indicated that the patients did not see any point in the addition of game-like elements, such as “I do not see additional value” or “not interesting.”

**Table 3:** Illustrative quotes of the patients.

Identified barriers and promoters	Indicative quote	Patients with a similar quote (n)
Grip on disease and better communication with physician	…*Improves interaction with my doctor, as the complaints I have had in the past month are now clearer.*	5
Disease activity	…*When my pain relapses, I would be more inclined to fill out the questionnaire.*	5
Technical problems	…*Technical problems prevented me from further usage.*	4
Respondent fatigue	…*It is the same (questionnaire) every time.*	4
App reminds patients of their disease	…*When I fill out the questionnaire, it makes me feel like a patient, I prefer not to feel like a patient this often.*	3

## Discussion

### Summary

This study shows the design, development, and evaluation of a smartphone app that allows patients with RA to monitor their disease activity off-site. This app was developed in line with the recommendations by the European League Against Rheumatism taskforce for development of mHealth apps, which were published after the current pilot studies were performed [30]. The pilot studies showed promising satisfaction (overall) and usability ratings; however, the app usage rates remain a challenge. Furthermore, patients indicated that they agreed with self-monitoring to be able to better allocate clinical consultations according to need. 

The overarching aim of the app is to reduce the frequency of clinic visits if the self-monitored disease activity is low, thereby reducing the health care burden for patients, and healthcare costs.We believe that the app is ready to evaluate these anticipated benefits in a randomized controlled trial, as the overall satisfaction and usability ratings were very promising. The NPS showed less positive results, with a negative and a neutral score, which may indicate that patients would not likely recommend the app to others. The discrepancy between the NPS and the overall satisfaction rating may be caused by the cultural differences in scoring. The Dutch or the Europeans tend to give less extreme scores compared to the Americans, and the NPS originated in the United States [31]. If 8 was also considered a promoter score instead of a neutral score and 6 as a neutral score instead of a negative score, both pilot studies would have had a positive NPS rating. The positive NPS rating would better match the overall satisfaction rating. We believe that the proposed implementation strategy is also supported by patients because patients in this study and in previous research studies acknowledge that apps could assist allocation of clinic visits according to need [32]. Furthermore, they approved of self-monitoring (27/31, 87%) and would like to skip hospital visits if the self-monitored disease activity is low (25/31, 81%). There were no concerns with regard to data privacy and security with this app, and a majority of the patients intended to keep using this app in the future.

Declining adherence is a challenge with our app and for medical apps in general. In any eHealth trial, a substantial proportion of the users drop out before completion or stop using the app [33-35]. The frequency of usage was previously evaluated in 2 apps for patients with RA; the median completion rates were 91% of the daily questionnaires over 3 months and 79% of the daily questionnaires over 6 months [36,37]. Our completion rates were lower; this could be (partly) due to the difference in the intended usage frequency (daily versus weekly). One review shows that more frequent intended usage predicts better adherence [33]. For now, it is unclear how often a patient has to be monitored to better target consultation according to need. Hypothetically, if one questionnaire per month would be needed, it might be recommendable to set the intended usage to once a week to make sure that sufficient questionnaires are collected. Even considering the limited usage, we did collect at least one questionnaire per patient. If we get at least one questionnaire per patient per month for a year, we will still have 4 times more updates on their disease activity than when patients visit the outpatient clinic every 3 months. In the qualitative part of our study, we reported several factors that could play a role in the declining usage, including a lack of symptoms, technical barriers, and respondent fatigue, which are endorsed by previous research [32]. Possible ways to increase usage would include providing shorter questionnaires or adaptive questionnaires, improving persuasive and gamified app designs, adding reminder notifications, and limiting technical problems [33,38,39]. Furthermore, as patients reported that more disease activity stimulated usage, it is possible that patients mainly use the app in case of impending flares. This could mean that although usage is low, no flares are missed. This hypothesis should be further examined in larger observational studies.

### Strengths

We performed 2 pilot studies with different qualitative and quantitative approaches to evaluate the app. This optimized our understanding of the patients’ perspectives toward the app and its purpose and gave insights into the overall functioning of the app. We think these data provide meaningful insights to aspiring medical app designers and rheumatologists who are considering to prescribe apps to specific patient populations. Furthermore, the overall strengths of our project are patient involvement in all stages of the app development and integration of the app with the existing Reade electronic medical record. Ultimately, we have developed a mobile app that facilitates easy data entry for patients, and visualization of that data for both patients and physicians. The repetitive collection of PROs with the app combined with statistics in the patients’ existing electronic medical record has enormous research potential. This has been recognized before, but this integration is often not accomplished [14,40,41].

### Limitations

As these were pilot studies, several limitations are present. First, the app is only available for patients of Reade, which limits generalizability. To improve this, we have, as a starting point, made our prototype available for other designers and health care centers. This will help others create a similar app. Second, it is possible that patients with enthusiasm for eHealth were more likely to participate. Therefore, it may be possible that the volunteers had above average technical skills and motivation to use the app. This warrants larger observational studies and controlled experiments in the future. Third, we cannot preclude that patients provided favorable feedback to the investigators. We did try to minimize this limitation by performing semistructured interviews and pilot studies with different researchers so that both had no previous relationships with the patients. Fourth, the semistructured interviews were rather short; however, after 9 interviews, no new opinions and data arose. We feel we have covered the most important opinions and experiences with patients. However, it could be possible that with longer interviews, more data would have been gathered. The last limitation is that patients who did not install the app were not included in the final questionnaire of the first pilot study—this may have led to an overestimation of the positive effects. To collect valuable information on the nonusers, we did purposefully include that specific group in the semistructured interviews to examine their barriers for adherence.

### Conclusion

Two pilot studies demonstrated that self-monitoring RA disease activity with the MijnReuma Reade app is feasible in terms of overall (patient) satisfaction and usability; however, the app usage rates remain a challenge. Patients acknowledged that the app had the potential to help them self-monitor their own disease so that they could reduce their frequency of clinic visits in case of low disease activity.

## Appendix

Multimedia Appendix 1 Statements of patients' perspectives.

Multimedia Appendix 2 Interview guide.

Multimedia Appendix 3 Heatmap of the patients' perspectives.

## Abbreviations

HAQ: Health Assessment Questionnaire
mHealth: mobile health
NPS: Net Promoter Score
PRO: patient-reported outcome
RAPID3: Routine Assessment of Patient Index Data 3
