# Supplementary material for: Feasibility of Self-Monitoring Rheumatoid Arthritis With a Smartphone App: Results of Two Mixed-Methods Pilot Studies
Source: JMIR Form Res. 2020 Sep 21;4(9):e20165. doi: 10.2196/20165 (PMC7536594; doi:10.2196/20165)
Supplement: Multimedia Appendix 1 [file formative_v4i9e20165_app1.docx]

Table 1. Overall Feedback

| Number | Statement |
| --- | --- |
| F1 | ‘I like that I can monitor my disease activity on my mobile phone’ |
| F2 | ‘With the app I experience more grip on my disease’ |
| F3 | ‘The app provides better insight in (fluctuations of) my disease activity’ |
| F4 | ‘I like that the app shows graphs of my disease activity over time’ |
| F5 | ‘I understood the numbers in the graphs after I read the explanation section’ |
| F6 | ‘I am fine with filling out my disease activity weekly when my disease becomes more active’ |
| F7 | ‘Filling out a questionnaire of 3-4 minutes is worth my time’ |
| F8 | ‘I would like to receive reminders to fill out the questionnaires’ |
| F9 | ‘I would like to be able to personalize when I receive reminders’ |
| F10 | ‘I would like to be able to add plain text notes about my disease’ |
| F11 | ‘I would like to be able to find a lot of information on my disease in the app’ |
| F12 | ‘I would like to receive personalized advice on the basis of my responses in the questionnaire’ |
| F13 | ‘I would like to be able to add personalized goals in the app (eg. being able to work, play sports, do social activities or sports)’ |
| F14 | ‘I would like the integration of a chat function with Reade’ |
| F15 | ‘The use of the app improves my preparation for a consultation with the rheumatologist’ |
| F16 | ‘If my disease activity is low, I would like the possibility that completing the questionnaires replaces an outpatient visit with my rheumatologist ’ |
| F17 | ‘I would like to be contacted by a rheumatology nurse when results of the questionnaire indicate increased disease activity’ |

Table 2. Privacy Statements

| Number | Statement |
| --- | --- |
| P1 | ‘It’s reassuring to know that the app stores information on my disease activity’ |
| P2 | ‘I am afraid that the information on my disease activity will fall into wrong hands’ |
| P3 | ‘I am worried that if I lose my phone, others will be able to see my disease activity’ |
| P4 | ‘I want to be able to decide who sees information regarding my disease activity’ |
| P5 | ‘I think the app is an infringement of my privacy’ |
| P6 | ‘I am fine with storing the information in my electronic medical record at Reade’ |
| P7 | ‘I am fine with a nurse following information my disease activity’ |
| P8 | ‘I am fine with a rheumatologist following information on my disease activity’ |
| P9 | ‘I want information on my disease activity only on my phone and not in my medical record’ |
