# Supplementary material for: Feasibility of Self-Monitoring Rheumatoid Arthritis With a Smartphone App: Results of Two Mixed-Methods Pilot Studies
Source: JMIR Form Res. 2020 Sep 21;4(9):e20165. doi: 10.2196/20165 (PMC7536594; doi:10.2196/20165)
Supplement: Multimedia Appendix 2 [file formative_v4i9e20165_app2.docx]

**Table 1.** Themes and matched questions in semistructured interviews.

| **Theme** | **Questions** |
| --- | --- |
| Subjective Quality | What did you think of the app? |
| Engagement | What where your reasons for (not) using the app? |
| Information | How was the app of value in your treatment? |
| Information | How do you feel about recording your disease scores over time in the app? |
| Aesthetics | How would your ideal version of the app look/what would you change? |
| Gamification | How would you feel about including game elements in the app? |
| Overall | Are there any other things that you would like to discuss regarding the app? |
