# Supplementary material for: Feasibility of Self-Monitoring Rheumatoid Arthritis With a Smartphone App: Results of Two Mixed-Methods Pilot Studies
Source: JMIR Form Res. 2020 Sep 21;4(9):e20165. doi: 10.2196/20165 (PMC7536594; doi:10.2196/20165)
Supplement: Multimedia Appendix 3 [file formative_v4i9e20165_app3.docx]

**Table 1. Heat map of Patients’ Perspectives.** The rows on the right indicate how the statements are scored by patients. Each cell is colorized based on the number of patients that rated the statement with the value on top of the row. The heat map ranges from yellow to blue, cells become more blue as more patients have rated the statement with that mark. The statements can be found in Multimedia Appendix 1.

**Table 2. Heat map ‘Privacy Statements’.** The rows on the right indicate how the statements are scored by patients. Each cell is colorized based on the number of patients that rated the statement with the value on top of the row. The heat map ranges from yellow to blue, the cell becomes more blue as more patients have rated the statement with that mark. The complete statements can be found in Multimedia Appendix 1.
